# Obesity by High-Fat Diet Increases Pain Sensitivity by Reprogramming Branched-Chain Amino Acid Catabolism in Dorsal Root Ganglia

**DOI:** 10.3389/fnut.2022.902635

**Published:** 2022-05-12

**Authors:** Nan Lian, Kaiteng Luo, Huijing Xie, Yi Kang, Kuo Tang, Peilin Lu, Tao Li

**Affiliations:** ^1^Department of Anesthesiology, National-Local Joint Engineering Research Centre of Translational Medicine of Anesthesiology, West China Hospital of Sichuan University, Chengdu, China; ^2^Laboratory of Mitochondria and Metabolism, West China Hospital of Sichuan University, Chengdu, China; ^3^Department of Radiology, Huaxi MR Research Center (HMRRC), Functional and Molecular Imaging Key Laboratory of Sichuan Province, West China Hospital of Sichuan University, Chengdu, China

**Keywords:** obesity, high-fat diet, pain behaviors, branched-chain amino acids, inflammation

## Abstract

Obesity is a significant health concern as a result of poor-quality diet, for example, high-fat diet (HFD). Although multiple biological and molecular changes have been identified to contribute to HFD-induced pain susceptibility, the mechanisms are not fully understood. Here, we show that mice under 8 weeks of HFD were sensitive to mechanical and thermal stimuli, which was coupled with an accumulation of branched-chain amino acids (BCAAs) in lumbar dorsal root ganglia (DRG) due to local BCAA catabolism deficiency. This HFD-induced hyperalgesic phenotype could be exacerbated by supply of excessive BCAAs or mitigated by promotion of BCAA catabolism *via* BT2 treatment. In addition, our results suggested that HFD-related pain hypersensitivity was associated with a pro-inflammatory status in DRG, which could be regulated by BCAA abundance. Therefore, our study demonstrates that defective BCAA catabolism in DRG facilitates HFD-induced pain hypersensitivity by triggering inflammation. These findings not only reveal metabolic underpinnings for the pathogenesis of HFD-related hyperalgesia but also offer potential targets for developing diet-based therapy of chronic pain.

## Introduction

High-fat diet (HFD), consisting of at least 35% of calories from fats, is correlated with increased risks of several chronic diseases, such as obesity, diabetes and cardiovascular diseases (1). Also, prolonged HFD intake is implicated in aberrant nociception and manifestations of allodynia (2). Although multiple biological and molecular changes have been identified to contribute to HFD-induced pain susceptibility, the mechanisms are not fully understood (3).

Branched-chain amino acids (BCAAs), including leucine, isoleucine, and valine, are essential amino acids for mammals and comprised of ∼ 20% of the amino acids incorporated into body protein (4). The rate of protein turnover, BCAA intake, and catabolism are key mechanisms in governing BCAA homeostasis in the body (5). In the BCAA catabolic pathway, BCAAs are first converted into branched-chain alpha-ketoacids (BCKAs) by branched-chain amino-transferase (BCAT) in a reversible reaction, followed by irreversible decarboxylation by branched-chain alpha-ketoacid dehydrogenase (BCKDH) complex, and eventually metabolized to acetyl-CoA or succinyl-CoA for oxidation in the tricarboxylic acid (TCA) cycle (4).

Epidemiological evidence shows that elevation of circulating BCAAs and related metabolites are strongly associated with insulin resistance and diabetes (6, 7). An accumulation of BCAAs in dorsal root ganglia (DRG) have also been observed in diabetic neuropathy, of which one third of patients will develop neuropathic pain (8), supporting a link between defective BCAA catabolism and chronic pain. Indeed, defective BCAA catabolism has been shown to correlate with inflammation in rodents and obese patients, and inflammation is believed to contribute to HFD-induced chronic pain (9, 10). Nonetheless, whether BCAA homeostasis regulates HFD-related pain behaviors is still elusive.

In the present study, we sought to elucidate the relationship between BCAA catabolism and pain susceptibility in HFD-fed mice. Our results reveal that excessive BCAAs in DRG is a culprit in HFD-induced pain susceptibility, and the findings may shed light on the diet-based treatment of chronic pain by targeting BCAA catabolism.

## Materials and Methods

### Mice

All animal experiments were approved by the Animal Ethics Committee of West China Hospital, Sichuan University, China. The animals received humane care in accordance with the *Guides for the Care and Use of Laboratory Animals* published by NIH. Male C57BL/6J mice were purchased from Dossy Experimental Animals Co. Ltd, Chengdu, China. Mice were maintained in a vivarium with a 12-hour light/dark cycle at 22*^o^*C and water was available *ad libitum*. The 4-week-old mice were randomly fed with high-fat diet (HFD), composed of 60% kcal per kg fat (D12492, Research Diets Inc, New Brunswick, NJ, United States), or chow diet (CD), composed of 11.85% kcal per kg fat (SWC9101, Xietong LTD, CN) for 8 or 16 weeks. The body weight and random blood glucose of mice were recorded weekly. For the measurement of blood glucose, approximately 5 μl of blood was collected *via* tail and analyzed with Blood Glucose Meter (Roche, Switzerland). At the end of the experimental protocol, mice were sacrificed to collect plasma and bilateral lumbar 4–5 (L4-5) DRG. Samples were quickly frozen in liquid nitrogen and stored until analysis.

### Glucose and Insulin Tolerance Tests

Glucose tolerance test (GTT) and insulin tolerance test (ITT) were performed after 8 weeks on HFD and CD, as described previously (11). Prior to ITT and GTT, mice were fasted for 4 or 6 h, respectively. After the measurement of fasting blood glucose, insulin (0.75 U/kg body weight) or D-glucose (2 g/kg body weight) were administrated through intraperitoneal injection. Blood glucose levels were determined at 15, 30, 60, 90, and 120 min by a Blood Glucose Meter (Roche, Switzerland).

### Branched-Chain Amino Acid or BT2 Administration

After 4 weeks on HFD, mice were randomly divided into three groups to receive water (Vehicle; Veh), 2% BCAA (weight ratio, isoleucine: leucine: valine = 0.8:1.5:1, Sigma Aldrich, St. Louis, MO, United States) or BT2. Compound BT2 (3,6-dichlorobenzo[b]thiophene-2-carboxylic acid, Santa Cruz Biotechnology, Santa Cruz, CA, United States), an inhibitor of branched-chain alpha-ketoacid dehydrogenase kinase (BCKDK), is diluted with solution (5% DMSO, 10% cremophor EL, and 85% 0.1 M sodium bicarbonate, pH 9.0) and administered by oral gavage at 40 mg/kg body weight per day.

### Behavioral Analyses

For evaluating mechanical allodynia, paw withdrawal thresholds in response to mechanical stimuli were measured by calibrated von Frey filaments (Stoelting Company, Wood Dale, IL, United States). Briefly, mice were placed in an individual Plexiglas chamber on an elevated mesh grid floor. After adaption for 1 h, von Frey filaments were used to stimulate the middle of the plantar surface of left and right hind paws. In the paradigm of the up-down method, test was initiated with a 0.16 g in the middle of the series force (0.008, 0.02, 0.04, 0.07, 0.16, 0.4, 0.6, 1.0, and 1.4 g). If a negative response occurred, the next stronger stimulus was applied; if a positive response appeared, the next weaker stimulus was chosen. The test was terminated when positive response (e.g., raising, retracting, rapidly swinging or licking feet reaction) was stimulated 3 times with an interval of 5 min by certain increasing irritation. We took the average value of filaments as mechanical threshold.

In the Hargreaves test, paw withdrawal latencies to thermal stimulation were examined with a radiant heat source (Model 37370; Ugo-Basile, Italy). In quiet room, mice were placed in a transparent organic chamber on a glass plate. After mouse adapting to circumstances, the plantar surface of each hind paw was stimulated by light source (IR = 35 mW/cm^2^). The light source was automatically turned off when hind paws moved and withdrawal latencies were recorded. Each plantar was repeated 3 times at 10 min intervals. To avoid damage to the hind paw, a cut-off time of 20 s was set up. Experimenters were blinded to the group allocation.

### Measurement of Branched-Chain Amino Acid Content

Plasma or DRG tissue samples were mixed into equal volume of acetonitrile. Then the supernatant was obtained by high-speed centrifugation and analyzed the BCAA levels by liquid chromatography-tandem mass spectrometry (1260-6460, Agilent, United States).

### RNA Isolation and Quantitative Real-Time PCR

Total RNA was isolated from frozen DRG tissues using the RNeasy Kit (Invitrogen, Thermo Fisher Scientific, Grand Island, NY, United States), and cDNA was synthesized using iScript cDNA Synthesis Kit (1708890, BIO-RAD, Hercules, CA, United States). qPCR was performed using the iTaq Universal SYBR Green Supermix (1725124, BIO-RAD, CA) with template cDNA and primers for each gene in a BIO-RAD CFX96 real-time PCR system. The relative mRNA expression levels were calculated by the ΔΔCt method and the results were normalized to β-actin levels. The primer sequences are listed as below.

*Il-6*: F-CCAAGAGGTGAGTGCTTCCC, R-CTGTTGTTCA GACTCTCTCCCT;

*Il-1*β: F-GCAACTGTTCCTGAACTCAACT, R-ATCTT TTGGGGTCCGTCAACT;

*Ccl3*: F-TTCTCTGTACCATGACACTCTGC, R-CGTGG AATCTTCCGGCTGTAG;

*Cxcl1*: F-CTGGGATTCACCTCAAGAACATC, R-CAGGG TCAAGGCAAGCCTC;

β*-actin*: F- GCAGGAGTACGATGAGTCCG, R-ACGCAGC TCAGTAACAGTCC.

### Western Blot

Frozen DRG tissues were lysed with RIPA buffer with protease inhibitor cocktail (Roche), PMSF and Na_2_VO_3_. Protein sample (5–20 μg) was separated by SDS–PAGE and transferred to a PVDF membrane. Membrane was blocked in 5% non-fat milk and incubated with primary antibodies at 4*^o^*C overnight with mild agitation, including anti-PP2Cm (DF4348, Affinity, San Francisco, CA, United States), anti-BCKDHA-E1α (#90198, Cell Signaling Technology, Danvers, MA, United States), anti-phosphor-BCKDHA-E1α (Ser293) (#40368, Cell Signaling Technology, Danvers, MA, United States), and anti-GAPDH (10494-1-AP, Proteintech, Rosamond, IL, United States) antibodies. After incubation with secondary antibodies (SA000001-2, Proteintech, Rosamond, IL, United States) at room temperature for 1 h, the signal intensities were visualized by an ECL Western blot detection kit (#P10100, ECM, Pleasant Prairie, WI, United States) and analyzed with Image J software (NIH).

### Branched-Chain Alpha-Ketoacid Dehydrogenase Activity Assay

The BCKDH complex activity in the DRG was analyzed as described previously (12). The BCKDH enzymatic activity was defined as the rate of formation of 1 μmol of NADH/min at 30°C. Both actual and total activities were measured; the former corresponded to *in vivo* dephosphorylated enzyme levels and the latter was obtained by treating the tissue extract with lambda protein phosphatase to fully dephosphorylate enzymes. The BCKDH activity is calculated as the percentage of actual activity relative to total activity.

### Hematoxylin and Eosin Staining

L4-5 DRG were fixed in 4% polyformaldehyde followed by dehydration in an ascending ethanol series. Tissues were embedded in paraffin and cut into 5 μm sections for Hematoxylin and Eosin (H&E) staining (ab245880, Abcam, Cambridge, United Kingdom). Morphological analysis was performed on three sections per DRG using OlyVIA digital software.

### Statistical Analysis

Statistical analysis was performed with GraphPad Prism 8 software. All data were presented as the mean ± SEM. Grayness of protein strips were analyzed by Image J software. Fold change of protein expression were analyzed with two-tailed unpaired Student’s *t*-tests. Body weight, blood glucose changes, and BCAA content were analyzed with two-way repeated measures analysis of variance (ANOVA). Behavioral time course data were analyzed with two-way ANOVA to determine overall differences between diet groups, with Bonferroni posttests to determine significance of differences between groups at each individual time point. The area of DRG cells, cell gaps, and the number of inflammatory cells in L4-5 DRG were counted and measured by the Image-Pro plus 6.0 pathological analysis software. The expression of inflammatory mediator was analyzed with one-way ANOVA with Dunnett’s posttest within each group. Differences were considered significant when *P* < 0.05.

## Results

### High-Fat Diet Induces Mechanical and Heat Hyperalgesia in Mice

**FIGURE 1:**
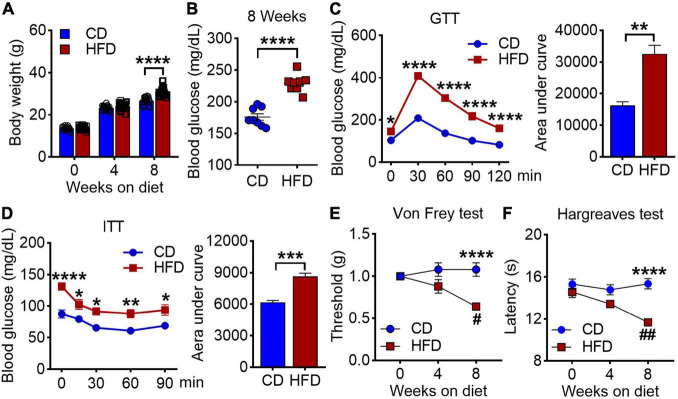
Effects of high-fat diet on body weight, blood glucose and pain behaviors in mice. **(A)** Body weights were measured at 0, 4 and 8 weeks on either chow diet (CD) or high fat diet (HFD) (*n* = 20–40). **(B)** Blood glucose levels were determined on 8 weeks of CD and HFD feeding (*n* = 4–8). **(C)** Glucose tolerance test (GTT) of mice at 8 weeks CD or HFD diet after 6 h fasting, and the area under curve (*n* = 20). **(D)** Insulin tolerance tests (ITT) of mice at 8 weeks CD or HFD diet after 4 h fasting, and the area under curve (*n* = 7). **(E,F)** Mechanical withdrawal thresholds **(E)** and withdrawal latencies to heat stimulus **(F)** after 4 and 8 weeks on CD or HFD (*n* = 5–15). Data are present as mean ± SEM. *P < 0.05, **P < 0.01, ***P < 0.005, and ****P < 0.001 for CD vs. HFD mice. ^#^*P* < 0.05, ^##^*P* < 0.01 for HFD mice at 4 weeks on diet vs. HFD mice at 8 weeks on diet.

To determine whether HFD affects nociceptive responses, we fed 4-week-old male mice with either standard CD or HFD for 8 weeks. As expected, mice under 8 weeks of HFD showed a greater body weight, higher blood glucose levels after overnight fasting, and impaired glucose tolerance as compared to the CD group (Figures 1A–D). By utilizing von Frey test and Hargreaves test, we found that the mice exhibited reduced nociceptive thresholds to both mechanical and heat stimuli after 8 weeks on HFD (Figures 1E,F). These results suggest that HFD lowers nociceptive thresholds and induces mechanical and thermal hyperalgesia.

### High-Fat Diet-Induced Hyperalgesia Is Coupled With an Accumulation of Branched-Chain Amino Acids in Dorsal Root Ganglia

**FIGURE 2:**
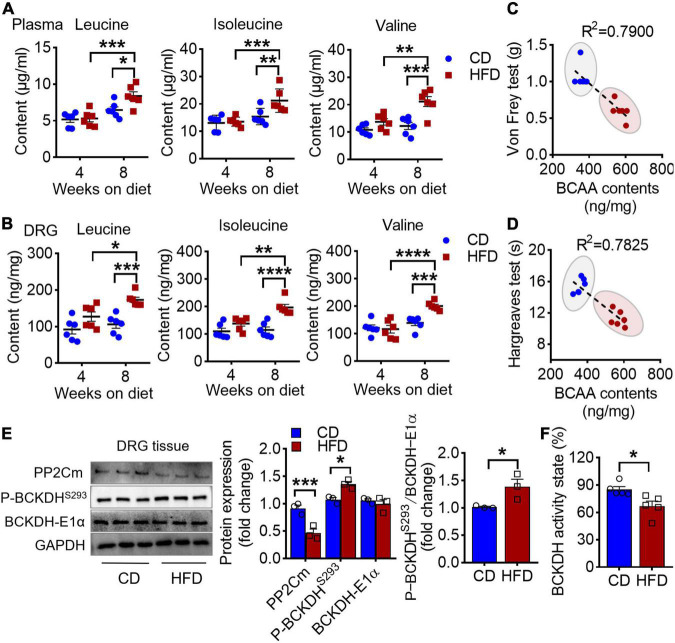
HFD-induced hyperalgesia is coupled with an accumulation of BCAAs in DRG. **(A)** BCAA (leucine, isoleucine, and valine) levels in plasma after 4 and 8 weeks feeding in CD (*n* = 6) and HFD mice (*n* = 6). **(B)** BCAA levels in L4-L5 DRG under 4 and 8 weeks of CD (*n* = 6) and HFD diet (*n* = 6). **(C,D)** The linear regression analysis between mechanical **(C)** or thermal **(D)** withdrawal thresholds and BCAA levels (leucine + isoleucine + valine) in L4-L5 DRG under 8 weeks of diet. **(E)** Western blots analysis of BCAAs catabolic enzymes, PP2Cm, P-BCKDH*^S293^* and BCKDH-E1α, in L4-L5 DRG after 8 weeks on CD (*n* = 3) or HFD (*n* = 3). **(F)** The BCKDH activity state in DRG after 8 weeks on CD (*n* = 3) or HFD (*n* = 3). Activity state means percentage of the BCKDH complex in the active dephosphorylated form. Data are shown as mean ± SEM. *P < 0.05, **P < 0.01, ***P < 0.005 and ****P < 0.001.

A strong association between disrupted BCAA homeostasis and diabetes has been repeatedly observed in human and rodent models (13). In line with it, we found an accumulation of plasma BCAAs in the mice on 8 weeks of HFD (Figure 2A). Moreover, an ∼2-fold increase in BCAA level was also observed in L4-L5 DRG, the hub that senses and transmits periphery stimuli to central nervous system (Figure 2B). Intriguingly, the DRG BCAA levels were linearly correlated with the graded decrease of the mechanical and thermal thresholds (Figures 2C,D and Table 1). BCKDH complex, the rate-limiting enzyme of the BCAA catabolic pathway, is activated by dephosphorylation *via* a mitochondrial localized 2C-type serine-threonine protein phosphatase (PP2Cm). Further study demonstrated that this increase of BCAAs in DRG was accompanied by a downregulation of protein abundance of PP2Cm, a hyperphosphorylation of BCKDH-E1α at Ser293 and a reduced BCKDH activity, suggesting that 8 weeks of HFD induces a defective BCAA catabolism in DRG (Figures 2E,F). Of note, with no pain behavioral changes, the mice on 4 weeks of HFD did not present an aberrance of BCAA levels. Together, these findings suggest that HFD-induced hyperalgesia is couple with a defective BCAA catabolism in DRG.

**TABLE 1:** The data of mechanical and thermal withdrawal thresholds and BCAA levels in DRG of every mouse, and the linear regression analysis.

Diet	Leucine (ng/mg)	Isoleucine (ng/mg)	Valine (ng/mg)	Von Frey (g)	Hargreaves (s)	Regression analysis
CD	115.0927	156.42936	95.7264	1.0	16.73	Von Frey: *R^2^* = 0.8195 *P* < 0.0001;
	71.07272	97.46744	154.21455	1.0	14.40	
	120.311799	114.58475	145.9364	1.0	16.3	
	81.42917367	137.70989	131.9812025	1.0	14.67	
	108.4733768	89.68346	154.8836364	1.4	14.63	
	141.52789039	92.93815	152.05744667	1.0	15.70	
HFD	176.4357579	177.7591365	226.4337147	0.6	12.10	Hargreaves: *R^2^* = 0.7825 *P* = 0.0001
	159.7703104	189.8461041	182.9511432	0.6	10.80	
	159.8991897	250.8975136	195.9405613	0.6	10.10	
	173.880632	198.5832559	192.499102	0.6	10.60	
	161.4239672	178.1349855	199.7653367	0.6	12.80	
	206.9810212	186.0674423	209.0355112	0.4	11.10	

### Branched-Chain Amino Acids Supplementation Exacerbates High-Fat Diet-Induced Hyperalgesia

**FIGURE 3:**
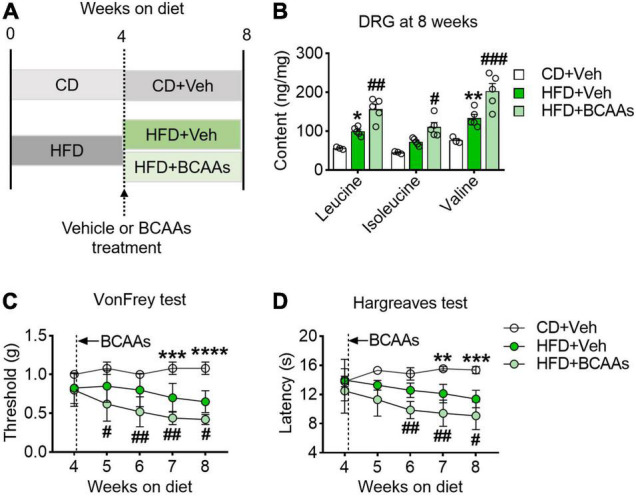
BCAAs supplementation aggravates pain behaviors in HFD-fed mice. **(A)** Schematic of 8-week HFD with BCAA supplementation paradigm. To demonstrate whether BCAAs play a causal role in HFD-induced hyperalgesia, mice were changed to a 2.2% BCAAs drinking water at 4 weeks. **(B)** BCAA levels in L4-L5 DRG after 8 weeks diet with or without BCAAs supplementation in CD + Veh (*n* = 3), HFD + Veh (*n* = 5) and HFD + BCAAs (*n* = 5). **(C,D)** Mechanical withdrawal thresholds **(C)** and withdrawal latencies to heat stimulus **(D)** at 8 weeks diet in CD + Veh (*n* = 5), HFD + Veh (*n* = 8–9) and HFD + BCAAs (*n* = 9–10). Data are shown as mean ± SEM. *P < 0.05, **P < 0.01, ***P < 0.005 and ****P < 0.001 for HFD + Veh vs. CD + Veh; ^#^*P* < 0.05, ^##^*P* < 0.01, ^###^*P* < 0.005, for HFD + Veh vs. HFD + BCAAs.

The above observation raised the question of whether BCAAs play a causal role in HFD-induced hyperalgesia. To study it, we supplemented BCAAs in the drinking water of the HFD-fed mice at 4 weeks, the time point having no hyperalgesic phenotype yet, and performed pain behavioral tests once a week during the following 4 weeks (Figure 3A). After 4 weeks of BCAAs and HFD co-feeding, the abundance of all the three BCAAs in the DRG was significantly increased as compared to the CD group and HFD-fed only group (Figure 3B). Von Frey test showed that simultaneous supply of BCAAs progressively and further reduced HFD-induced downregulation of mechanical withdrawal thresholds; hypersensitivity to mechanical stimuli was observed in the HFD-fed mice (5 weeks) with 1 week of BCAAs supplementation, and the response was comparable to that of the mice under 8 weeks of HFD only (Figure 3C). A similar effect of BCAAs in reducing thermal paw withdrawal latencies was also observed in the Hargreaves test (Figure 3D). Therefore, these results suggest that supply of BCAAs increases their abundance in DRG and exacerbates HFD-induced hyperalgesia.

### Promoting Branched-Chain Amino Acid Catabolism Prevents and Rescues High-Fat Diet-Induced Hyperalgesia

**FIGURE 4:**
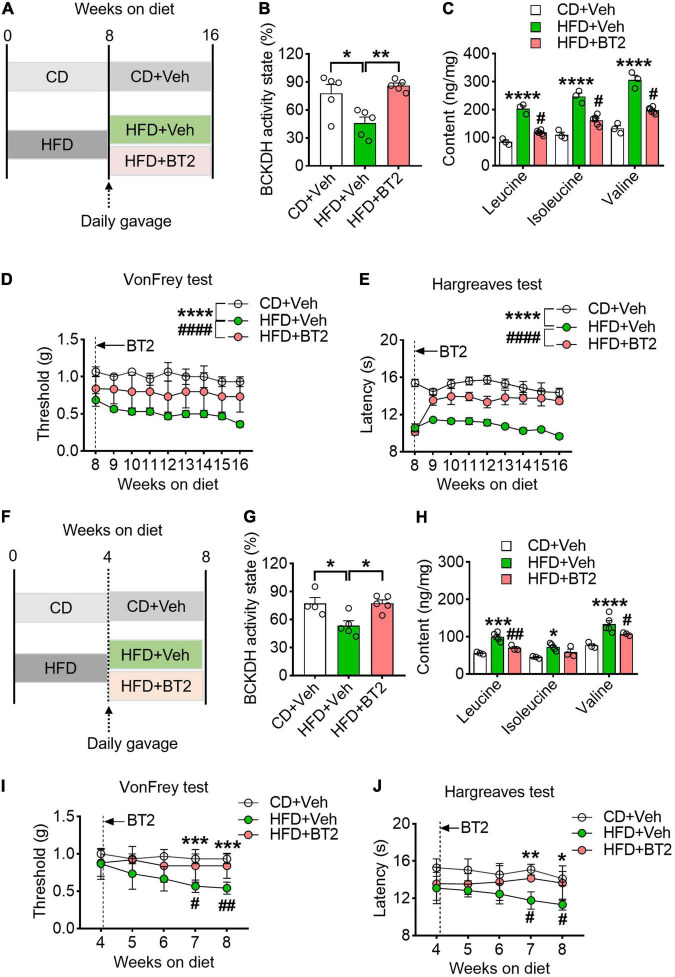
Promoting BCAA catabolism prevents and rescues HFD-induced hyperalgesia. **(A)** Schematic of the BT2 diet-reversal paradigm, showing mice after 8 weeks HFD either continuing the HFD or receiving BT2 for 8 weeks. **(B,C)** The BCKDH activity state and BCAA levels in L4-L5 DRG at 16 weeks on HFD with or without BT2 supplementation in CD + Veh (*n* = 3), HFD + Veh (*n* = 3), and HFD + BT2 (*n* = 6). **(D,E)** Mechanical withdrawal thresholds **(D)** and withdrawal latencies to heat stimulus **(E)** from 8 to 16 weeks diet in CD + Veh (*n* = 5–6), HFD + Veh (*n* = 4–6) and HFD + BT2 (*n* = 5–6). **(F)** Schematic of the BT2 diet-reversal paradigm, to demonstrate whether promoting BCAA catabolism delay the development of hyperalgesia under HFD, the 4-week-HFD mice were supplemented with BT2. **(G,H)** The BCKDH activity state and BCAA abundance in L4-L5 DRG at 8 weeks on HFD with or without BT2 supplementation in CD + Veh (*n* = 3), HFD + Veh (*n* = 5), and HFD + BT2 (*n* = 3). **(I,J)** Mechanical withdrawal thresholds **(I)** and withdrawal latencies to heat stimulus **(J)** from 4 to 8 weeks in CD + Veh (*n* = 6), HFD + Veh (*n* = 6), and HFD + BT2 (*n* = 5–6). Activity state means percentage of the BCKDH complex in the active dephosphorylated form. Data are present as mean ± SEM. *P < 0.05, **P < 0.01, ***P < 0.005 and ****P < 0.001 for HFD + Veh vs. CD + Veh; ^#^*P* < 0.05, ^##^*P* < 0.01 and ^####^*P* < 0.001 for HFD + Veh vs. HFD + BT2.

To further determine whether accumulation of BCAAs caused by defective catabolism was responsible for the pain phenotype, we sought to enhance BCAA catabolism in the HFD-fed mice *via* daily oral gavage of BT2, an activator of BCKDH, for 8 weeks (40 mg/kg/day) (Figure 4A). After treatment, the suppressed BCKDH activity in DRG caused by HFD was increased by BT2, and the excessive BCAAs were also largely eliminated (Figures 4B,C). The mechanical withdrawal thresholds and thermal paw withdrawal latencies of the HFD-fed mice were also upregulated throughout the course of treatment (Figures 4D,E). Notably, along with the decreased BCAA contents in DRG, both nociceptive responses of the HFD mice were normalized to the CD levels at the end of the 8-week treatment (Figures 4B–E). We next asked if promoting BCAA catabolism delayed the development of hyperalgesia under HFD, and thus supplemented BT2 to the 4-week-HFD mice (Figure 4F). We found that, along with increased BCKDH activity, the accumulation of BCAAs in DRG was mitigated by BT2, and the progressive decrease in nociceptive thresholds to mechanical and heat stimulations was also prevented (Figures 4G–J). Taken together, these data suggest that enhancing BCAA catabolism prevents the emerge of pain phenotypes and rescues established hyperalgesia under HFD.

### High-Fat Diet-Induced Accumulation of Branched-Chain Amino Acids Evokes a Pro-inflammatory Response in Dorsal Root Ganglia

**FIGURE 5:**
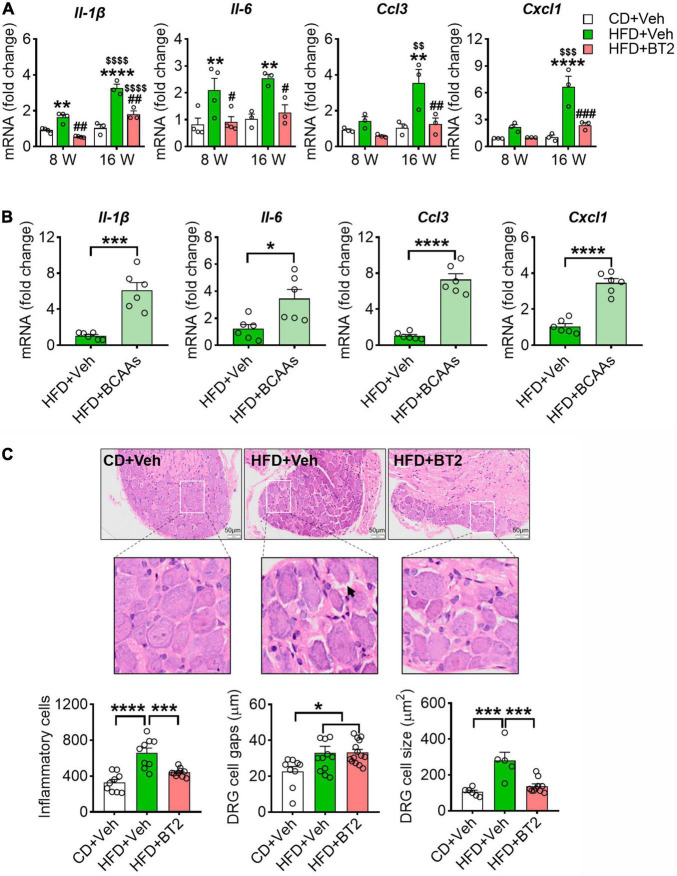
HFD-induced accumulation of BCAAs evokes a pro-Inflammatory response in DRG. **(A)** The relative mRNA expression of Il-1β, Il-6, Ccl3 and Cxcl1 (to β-actin) at 8 and 16 weeks in L4-L5 DRG of CD + Veh (*n* = 3-4), HFD + Veh (*n* = 3-4), and HFD + BT2 (*n* = 3-4) mice. Data are present as mean ± SEM. **(B)** The relative mRNA expression of Il-1β, Il-6, Ccl3 and Cxcl1 (to β-actin) on 8 weeks diet in L4-L5 DRG of HFD + Veh (*n* = 6) and HFD + BCAAs (*n* = 6) mice. **(C)** Representative images of HE-stained L4-L5 DRG section under 16 weeks of diet in CD + Veh, HFD + Veh, and HFD + BT2 mice. 200X magnification, bar indicating 50 μm. The cell gaps, area of ganglion cells, and number of inflammatory cells in L4-L5 DRG on 16 weeks diet of CD + Veh (*n* = 6–9), HFD + Veh (*n* = 5–11), and HFD + BT2 (*n* = 10–14). Data are present as mean ± SEM. *P < 0.05, **P < 0.01, ***P < 0.005, and ****P < 0.001 for HFD + Veh vs. CD + Veh or indicated comparisons; ^#^*P* < 0.05, ^##^*P* < 0.01 and ^###^*P* < 0.005 for HFD + Veh vs. HFD + BT2. ^$$^*P* < 0.01, ^$$$^*P* < 0.005 and ^$$$$^*P* < 0.001 for HFD + Veh at 8 weeks vs. HFD + Veh at 16 weeks, HFD + BT2 at 8 weeks vs. HFD + BT2 at 16 weeks.

We hypothesized that BCAA-modulated pain behavior in HFD mice is associated with an altered inflammatory status in DRG. To test it, we isolated the mouse DRG at the end of experiment and measured the pro-inflammatory cytokines/chemokines. In line with previous reports, 8 weeks on HFD boosted the mRNA expression of pro-inflammatory cytokines/chemokines in DRG, including *Il-1*β, *Il-6*, *Ccl3*, and *Cxcl1* (Figure 5A). This local inflammation in DRG was persisted or aggravated at 16 weeks (Figure 5A). Strikingly, promoting BCAA catabolism *via* BT2 treatment was able to abolish the pro-inflammatory effect of HFD on DRG at both time points (Figure 5A). By contrast, supplementation of BCAAs in the HFD-fed mice further upregulated the mRNA levels of these cytokines/chemokines, suggesting that excessive BCAAs in DRG may synergize with HFD to evoke local inflammation (Figure 5B). In addition, the H&E staining images showed a more severe inflammatory infiltration in DRG after 16 weeks of HFD feeding, and the cross-sectional area of ganglion cells and the gap between cells were also increased. All these pathological changes could be mitigated by 8 weeks of BT2 treatment (Figure 5C). Taken together, these results suggest that excessive BCAAs lead to a pro-inflammatory status in DRG, which might contribute to the HFD-induced hyperalgesic phenotype.

## Discussion

In the present study, we demonstrated that HFD-induced mechanical and thermal hyperalgesia is couple with a defective BCAA catabolism in DRG. This hyperalgesic phenotype could be exacerbated by supply of extra BCAAs or mitigated by promotion of BCAAs catabolism *via* BT2 treatment. In addition, our results suggest that BCAAs-regulated inflammatory status in DRG is responsible for the HFD-related pain behaviors. These findings, therefore, not only reveal metabolic underpinnings for the pathogenesis of HFD-related hyperalgesia but also offer potential targets for developing diet-based therapy of chronic pain.

How obesity regulates pain manifestation is still under debate (14). The findings in human studies comparing nociception in obese and lean individuals are seemingly paradoxical; some studies reporting lower thresholds (15, 16), while others reporting obesity-associated hypoalgesia (17). In HFD-fed rodent models, studies find either decreased response thresholds or no alterations under innocuous mechanical stimuli (18–20), and for the noxious thermal stimuli, studies present longer latencies, unaltered responses, or hyperalgesia in comparison to lean controls (21–23). In most studies using subacute and chronic pain models, including subcutaneous inflammation, arthritis and perineural inflammation, decreased thresholds and/or prolonged pain manifestations were reported in obesity models (24–26). In the present study, we found that HFD-fed obese mice displayed significant mechanical allodynia and thermal hyperalgesia, supporting the notion that obesity is associated with abnormal pain manifestation. In our opinion, in addition to genetic factors of different mouse strains, the time course of the pathology might account for some of the discrepancies observed across studies, because that a prolonged inflammation could lead to structural/functional alterations in the peripheral nerves and potentially blunt the perception of pain (2).

Accumulating evidence shows that circulating BCAA levels are elevated in obese humans and rodents, emerging as a predictor for the future risk of diabetes (27). The elevated BCAA levels in obese and/or diabetic individuals could be attributed to defective BCAA catabolism, as corroborate by the findings that BCAA catabolic enzymes are decreased in the obese *ob*/*ob* mice and *fa*/*fa* rat as well as obese humans (28), and that increasing BCAA catabolism by BT2 attenuates insulin resistance in ob/ob mice (13). The exact mechanisms responsible for the obesity-related BCAA dysregulation remain poorly understood, but may be attributed to the following two reasons: 1) the impaired central nerves system insulin signaling under obesity (28); 2) HFD-induced hyperglycemia which inhibits CREB-stimulated KLF15 transcription resulting in downregulation of enzymes in the BCAA catabolism pathway (29). In turn, the increased circulating BCAA levels experienced by obese individuals may lead to hyperactivation of the mTOR pathway that worsens systemic insulin resistance and KLF15 transcription, thus forming a vicious cycle.

Our study identified BCAA catabolism in DRG as a key regulatory point through which HFD modulates pain sensitivity. Unlike most of the amino acids, which are degraded in the liver, BCAAs are oxidized extensively in extra-hepatic tissue, although the significance of such a metabolic pattern has not been completely understood (5). The rate-limiting step in BCAA catabolism is BCKA decarboxylation in the mitochondria catalyzed by the BCKDH complex. PP2Cm is responsible for the dephosphorylation of the E1a subunit (Ser293) of the BCKDH, and hence activation of the complex (30). Here, we report that 8 weeks of HFD feeding impairs BCAA catabolism in mouse DRG, as evidenced by decreased protein expression of PPC2m and increased phosphorylation of BCKDH, and the resulted higher level of BCAAs in DRG is closely correlated with the sensitivity of mice to mechanical and thermal stimuli. Moreover, the effects of HFD on pain behaviors can be overridden by promotion of BCAA catabolism and exacerbated by supply of extra BCAAs, our results thereby indicate an emerging link between BCAA homeostasis in DRG and pain sensitivity. Similar downregulation of BCAA catabolism has also been reported in subcutaneous adipose of the HFD-fed mice (31). However, the current study did not address the mechanism by which HFD impairs BCAA catabolism. It could be due to HFD-induced elevation of blood glucose, for that high glucose has been shown to inhibit CREB stimulated KLF15 transcription resulting in downregulation of enzymes in the BCAA catabolism pathway (29). Further study is required to unveil the underlying regulatory machinery.

In line with a prevailing notion that obesity is a low-grade inflammatory condition (32), we found increased expression of pro-inflammatory cytokines/chemokines in mouse DRGs after 8 weeks of HFD feeding. It has been reported that *Il-1*β and *Ccl3* act as key neuroinflammatory regulators that directly increase the excitability of primary sensory neurons, prolong neuroinflammation and promote activation of the downstream cellular oxidative stress, facilitating neuropathic pain behavior (33–36). Cxcl1 can promote central sensitization which may also lead to chronic pain (37). Intriguingly, our results show that BCAA homeostasis plays a vital role in modulation of these changes. Along with reduced pain manifestation, promoting of BCAA catabolism by BT2 suppressed the pro-inflammatory status in DRG, while supplementation of BCAAs on top of HFD further enhanced the expressions of these cytokines/chemokines. Recent studies have suggested that increased BCAA abundance could activate mTORC1, upregulate the NF-κB signaling pathway, and boost the release of pro-inflammatory cytokines (38). Excessive BCAAs could also induce ROS production, and subsequently activate the Nod-like receptor pyrin domain containing 3 (NLRP3) inflammasome to produce IL-1β and IL-18 (39, 40). However, our study does not exclude the possibility that other forms of BCAAs-related damage also contributed to the pathogenesis, such as oxidative stress, calcium overload, and lipid peroxidation toxicity (41, 42). Nevertheless, our data propose a previously unappreciated effect of BCAA homeostasis on DRG inflammation and obese/HFD related pain hypersensitivity.

## Conclusion

The present study demonstrates that defective BCAA catabolism in DRG facilitates HFD-induced pain hypersensitivity by triggering inflammation. The present study unveils a critical role of BCAA catabolism in HFD-induced hyperalgesic phenotype, which may serve as a basis for the pathogenesis, as well as a target for therapy.

## Data Availability Statement

The raw data supporting the conclusions of this article will be made available by the authors, without undue reservation.

## Ethics Statement

The animal study was reviewed and approved by Animal Ethics Committee of West China Hospital, Sichuan University, China.

## Author Contributions

TL and PL conceived the project idea, design the protocol, and wrote the manuscript. NL and KL performed the experiments. NL, KL, HX, and KT performed the data analysis. All authors contributed to the article and approved the submitted version.

## Conflict of Interest

The authors declare that the research was conducted in the absence of any commercial or financial relationships that could be construed as a potential conflict of interest.

## Publisher’s Note

All claims expressed in this article are solely those of the authors and do not necessarily represent those of their affiliated organizations, or those of the publisher, the editors and the reviewers. Any product that may be evaluated in this article, or claim that may be made by its manufacturer, is not guaranteed or endorsed by the publisher.
